# Design and evaluation of EphrinA1 mutants with cerebral protective effect

**DOI:** 10.1038/s41598-017-02091-7

**Published:** 2017-05-15

**Authors:** Yuanjun Zhu, Yuanqing Gao, Danping Zheng, Mengyang Shui, Kuai Yu, Xiaoyan liu, Yuan Lin, Li Su, Wenxing Yang, Yinye Wang

**Affiliations:** 1 Department of Molecular and Cellular Pharmacology, School of Pharmaceutical Sciences, Peking University Health Science Center, Beijing, China; 2 State Key Laboratory of Bioactive Substances and Function of Natural Medicine, Institute of Materia Medica, Chinese Academy of Medical Sciences and Peking Union Medical College, Beijing, China; 3 Center of Medical and Health Analysis, Peking University Health Science Center, Beijing, China; 4 Department of Organismic and Evolutionary Biology, Center for Brain Science, Harvard University, Cambridge, MA USA

## Abstract

The activation of EphA2 receptor by its natural ligand EphrinA1 causes blood brain barrier dysfunction, and inactivation of EphA2 reduces BBB damage in ischemic stroke. Thus, EphA2 targeted antagonists may serve as neuroprotective agents. We engineered four mutants of EphrinA1, EM1, EM2, EM3 and EM4, respectively. The computational analysis showed that these four mutants were capable of interacting with EphA2. Their potential neuroprotective effects were examined in mouse focal ischemia/reperfusion (I/R) model. EM2 exhibited strong neuroprotective effects, including reduced brain infarct volume, neuronal apoptosis, cerebral edema, and improved neurological scores. The EM2-mediated protection was associated with a comparative decrease in BBB leakage, inflammatory infiltration, and higher expression levels of tight junction proteins, such as zonula occludens-1 and Occludin. I/R-induced high expression of Rho-associated protein kinase 2 (ROCK2) was down-regulated after EM2 treatment. Moreover, EM2 reduced agonist doxazosin-induced EphA2 phosphorylation and cells rounding in PC3 cells, indicating EphA2-antagonizing activity of EM2. These finding provided evidences of the neuroprotection of EphA2 antagonist and a novel approach for ischemic stroke treatment. These results also suggested that a receptor agonist can be switched to an antagonist by substituting one or more relevant residues.

## Introduction

Ischemic stroke is a major cause of death and disability worldwide^1^. Tissue damage following cerebral ischemia is mediated by multiple pathophysiological mechanisms. Not only neurons but also all other components of the neurovascular unit (NVU), consisting of glia, endothelium, pericytes and basal membranes, are involved in ischemic injury^2, 3^. The disruption of blood brain barrier (BBB), an important component of NVU, is thought to play a critical role in the pathophysiology of ischemia/reperfusion (I/R). Cerebral ischemia-induced increase in permeability of BBB further aggravates brain injury and affect prognosis of cerebral infarction^4–6^. Therefore, protecting BBB is a valuable strategy in stroke treatment.

Erythropoietin-producing hepatocellular receptors (Eph receptors) are the largest subfamily of receptor tyrosine kinases^7^. The Eph/ephrin (receptor–ligand) system plays an important role in a range of chronic and regenerative diseases, by influencing cell behavior through signaling pathways, resulting in modification of the cell cytoskeleton and cell adhesion^8, 9^. It has been shown previously that EphA2 (Eph type-A receptor 2) deletion (EphA2^−/−^)^10^ in mice can markedly attenuate BBB damage as evidenced by reduced brain edema, matrix metallopeptidase-9 (MMP-9) expression, infiltration of peripheral immune cells, and increased expression of tight junction protein zonula occludens-1 (ZO-1)^11^. In addition, inactivation of EphA2 by RNAi promoted tight junction formation in human brain microvascular endothelial cell line (HBMEC)^12^. Hence, targeted blockage of EphA2 activation may protect BBB in ischemic stroke.

In the present study, we aim to engineer EphA2 antagonists based on EphrinA1. Four EphrinA1 mutants were constructed, and their activities were examined *in vitro* and *in vivo*. We demonstrated for the first time that systemic delivery of a protein with EphA2-antagonizing activity results in a significant neuroprotection in cerebral ischemia.

## Results

### Design and construction of EphrinA1 mutants as EphA2 antagonist

**Figure 1:**
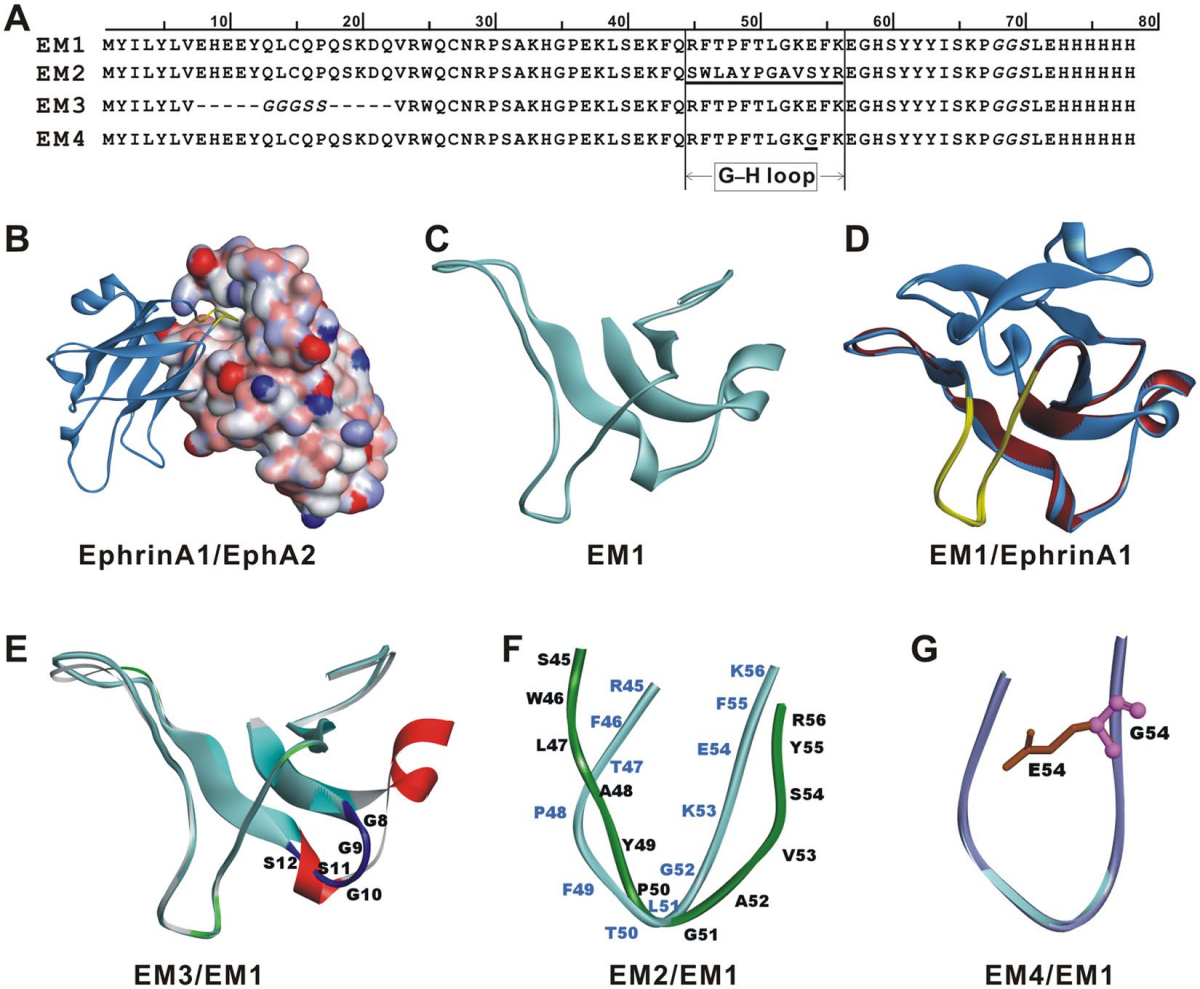
Protein sequences and the molecular models of EphrinA1 mutants. (**A**) The mutated amino acids in G–H loop are underlined and the flexible linker residues are shown in italics. (**B**) Overview of EphrinA1-EphA2 complex (PDB ID: 3HEI). EphrinA1 is in solid ribbon and EphA2 is in surface model. (**C**) The homology model of EM1. (**D**) EM1 (colored in red) superimposes with EphrinA1 (colored in medium blue). The G–H loop is highlighted in light yellow. (**E**) Superposition of EM3 with EM1. The α-helix (colored in red)-deleted EM1 is linked by GGGSS linker (colored in blue) in EM3. (**F**) Structure-sequence alignment of the crucial loop between EM1 (colored in light cyan) and EM2 (colored in green). (**G**) Single E54G mutation in G–H loop of EM4.

According to the structure of EphrinA1/EphA2 complex (PDB ID: 3HEI), the G–H loop (F108–K121) of ligand EphrinA1 inserted into a groove (formed among α helix D77–G84, α-helix V125–R132, and β-sheet W25–C43) of receptor EphA2 (Fig. 1B), which forms extensive intermolecular interaction^13^. Thus, we engineered four EphrinA1 mutants, EM1, EM2, EM3, and EM4 (Fig. 1), to obtain potential antagonists of EphA2. EM1 contains the G–H loop domain and its supporting scaffold, comprising 68 amino-acid residues of EphrinA1 (Q66–I133) and a 6× His-tag (Fig. 1A,C,D). We then constructed EM2 based on EM1, in which the loop residues RFTPFTLGKEFK was substituted by SWLAYPGAVSYR (Fig. 1A,F)^14^. EM3 was engineered by deleting the α-helix (E73–Q87)^15^ of EM1 and connecting the two ends by a short flexible linker (Fig. 1A,E). Structural studies also show that electrostatic interaction between E119 of EphrinA1 and R103 of EphA2 is closely related to the activation of EphA2 receptor^13^. Thus, the fourth variant EM4 (Fig. 1A,G) was designed by mutating the crucial residue E54 of EM1 (E119 of EphrinA1) to be glycine. The structures of the four mutants were homologically modeled by MODELER program, and then subjected to CHARMM energy minimization and further verified by Profiles-3D and Ramachandran plot programs. The model with the highest score (one of twenty) was selected for further investigation. The conformational changes were analyzed by molecular dynamics, and it showed that the loop residues were flexible in favor of interacting with EphA2 pocket, while the less fluctuation of the scaffold contributed to the structural stability of the whole protein (data not shown).

**Figure 2:**
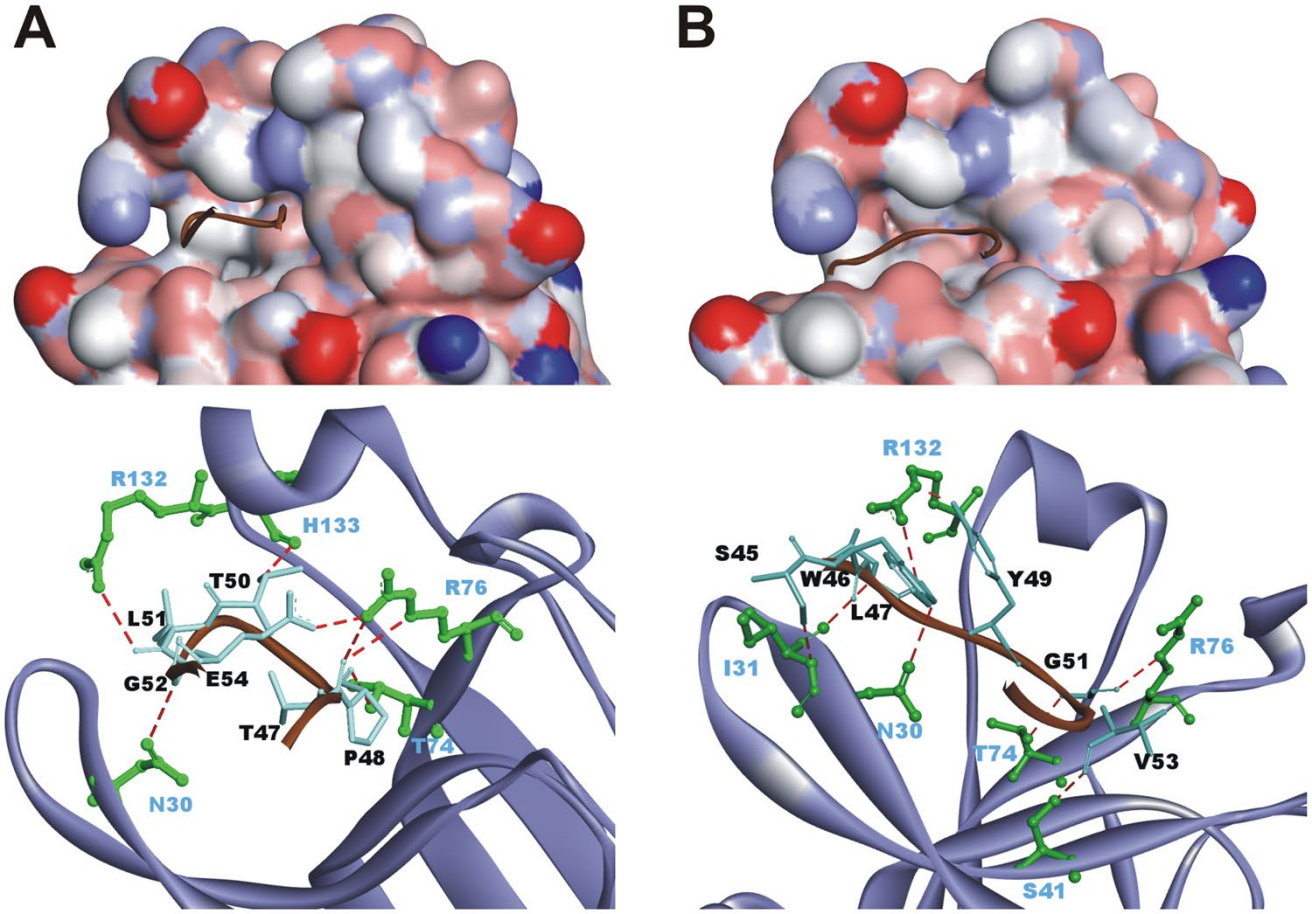
Computational analysis of EphA2-EphrinA1 mutant interaction. Overview (top) and the detailed intermolecular H-bond networks (bottom) between the inserting-loop and the groove of EphA2. The interaction between EM1 (**A**), or EM2 (**B**) and EphA2 was obtained by protein-protein docking analysis. Protein structure is shown as surface and/or ribbon model. The interacting residues of EphrinA1 variant and EphA2 are shown in sticks and ball-and-sticks, respectively, the H-bonds are shown in dashed line (red).

Molecular docking analysis was used to determine whether these EphrinA1 mutants could interact with EphA2 receptor. The G–H loop of EM1 could insert into the active pocket of EphA2 (Fig. 2A), quite similar with that between EphrinA1 and EphA2 (Fig. 1B). The SWL loop of EM2 was able to interact with EphA2, forming strong intermolecular hydrogen bonds (H-bond) (Fig. 2B). In addition, EM3 and EM4 showed similar binding model with EM1, except that the H-bond between E54 and R76 no longer existed in the binding of EM4 to EphA2. The inter-molecular interaction energy between EM1, EM2, EM3, EM4 and EphA2 reaches to −194.2 kcal/mol, −201.3 kcal/mol, −178.5 kcal/mol, and −192.1 kcal/mol, respectively.

The recombinant proteins were over-expressed as insoluble inclusion bodies when induced by IPTG at 37, 28, or 20 °C (Fig. S1A). After refolding by dilution method, these mutants were purified by Ni-NTA affinity chromatography (Fig. S1B). SDS-PAGE analysis showed that the purity of recovered target proteins was >85–90%.

### Protection of EphrinA1 mutants against transient cerebral ischemic injury

**Figure 3:**
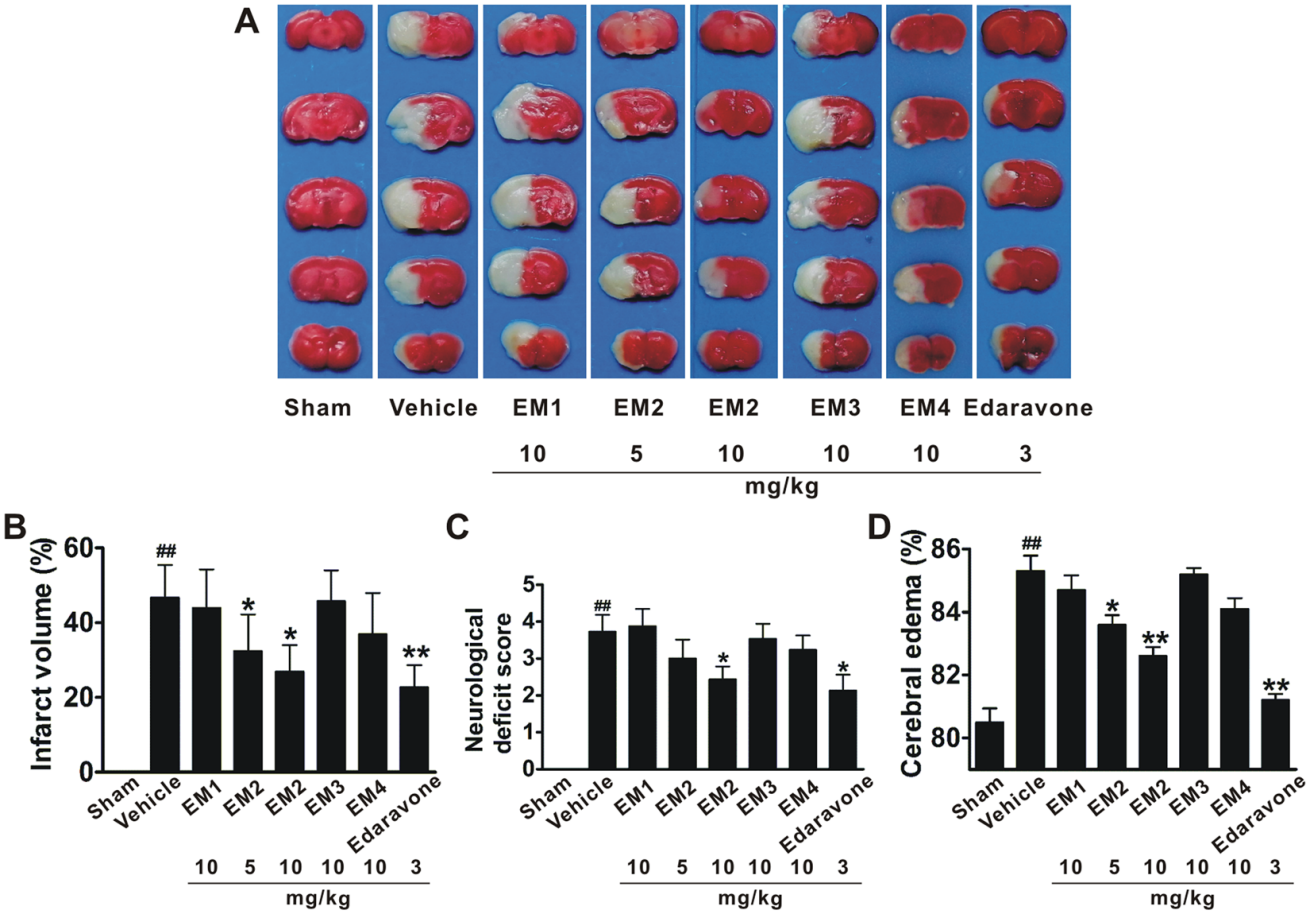
EphrinA1 mutants protect mice against I/R cerebral damage. (**A**) Representative TTC-stained brain coronal sections. Quantitative analysis of cerebral infarct volumes (**B**), neurological deficit scores (**C**), and cerebral edema (**D**). All data are presented as means ± S.E.M. (n = 10–12). ^##^ *p* < 0.01 compared with sham group. *p < 0.05, **p < 0.01 compared with vehicle group.

To investigate whether these recombinant EphrinA1 mutants can protect the brain against ischemic damage, we examined infarct volumes and neurological deficits in a mouse model of transient focal cerebral ischemia. The initial dosage of recombinant proteins was 10 mg/kg according to previous researches^16–18^. Vehicle-treated mice developed severe injuries with infarct volumes; while EM2 significantly reduced the infarct size and improved behavior score (Fig. 3A–C). However, the animals treated with EM1 or EM3 did not show any improvement in cerebral infarct and neurological scores (Fig. 3B,C). In addition, the EM4-treated mice showed a trend towards a reduction of infarction and neurological deficits (Fig. 3B,C).

We further examine whether EphrinA1 proteins can alleviate cerebral edema in I/R mice. Cerebral I/R significantly increased the water content of the brain, and EM2 prevented this increase, indicating a consistent and significant correlation with the infarct size (Fig. 3D). EM4-treated mice also showed a trend of reduction in brain water content, while the mice treated with EM1 or EM3 showed no obvious effect (Fig. 3D). Furthermore, EM2 reduced the infarct area, improved neurological score and decreased brain water content at a smaller dose of 5 mg/kg (Fig. 3). These data indicated that EM2 has a protective effect against mice focal cerebral ischemic injury.

### Effect of EM2 on cerebral apoptosis in ischemic brain

**Figure 4:**
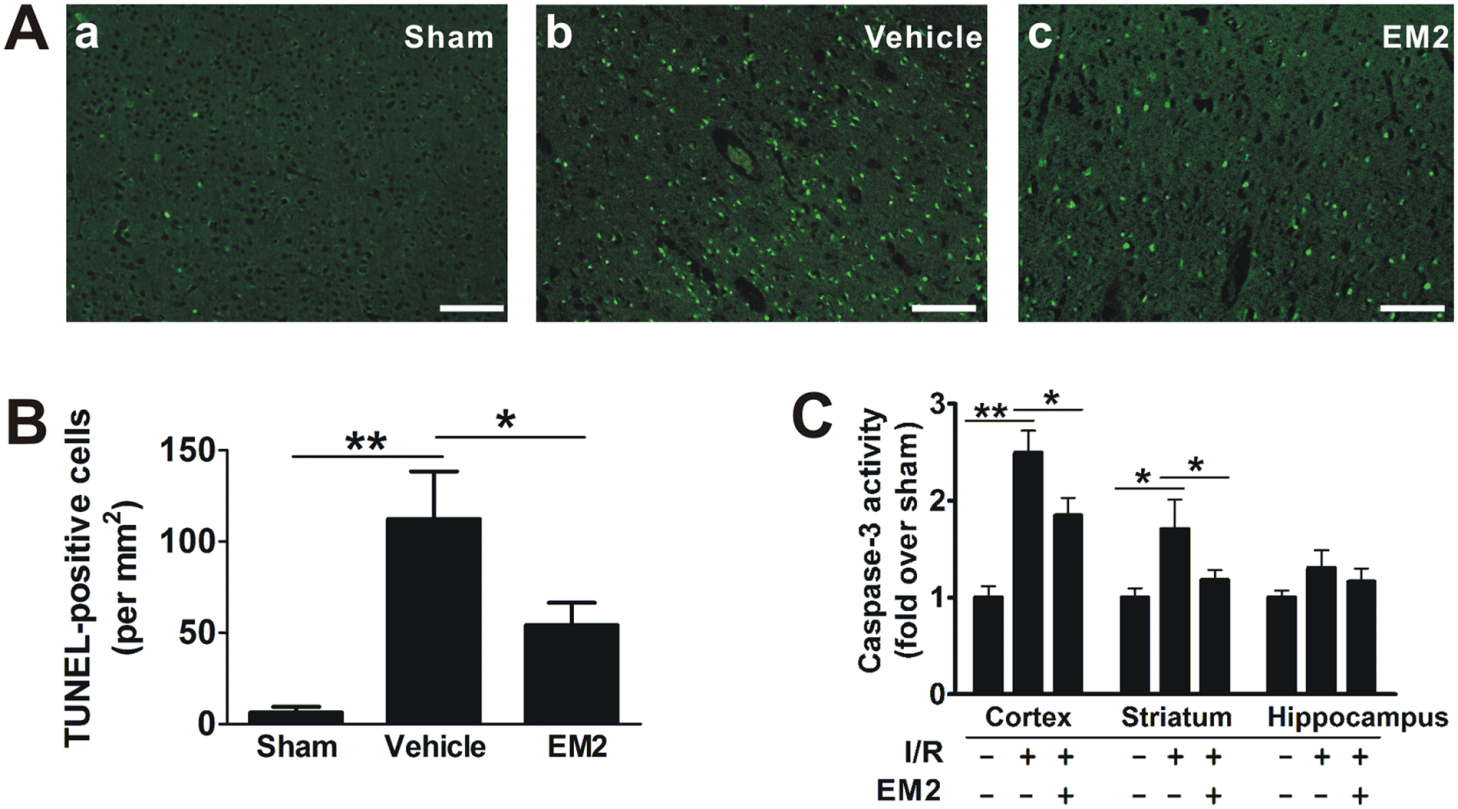
EM2 reduces ischemia-induced neuronal cell apoptosis and caspase-3 activation. (**A**) Representative photographs show TUNEL staining cells within the cortical ischemic penumbra area. (**B**) Quantification of the number of TUNEL-positive cells shown in *A* (n = 3). (**C**) Colorimetric detection of caspase-3 activity in different regions of brain (n = 3). All data are means ± S.E.M. *p < 0.05, **p < 0.01 compared with indicated group. Scale bar = 100 μm.

To explore the cellular mechanisms underlying the neuroprotective effect of EM2, we next evaluated the degree of apoptotic death using TUNEL staining and caspase-3 activity colorimetric assay. The number of apoptotic cells in the cortex was significantly reduced in EM2-treated (10 mg/kg) animals, compared with vehicle-treated group (Fig. 4A and B). Then we examined the caspase-3 activities in different zones of brain. The caspase-3 activity in the cortex of vehicle-treated mice was found to be significantly increased, which could be partly restored by EM2 treatment (Fig. 4C). The same tendency was seen in the striatum where EM2 treatment reduced apoptosis after 24 hours’ reperfusion. In the hippocampus, there was no significant change of caspase-3 activities among the sham, vehicle and EM2 groups. These data collectively demonstrated that EM2 reduces I/R-induced cerebral apoptosis.

### Effect of EM2 on BBB damage in ischemic brain

**Figure 5:**
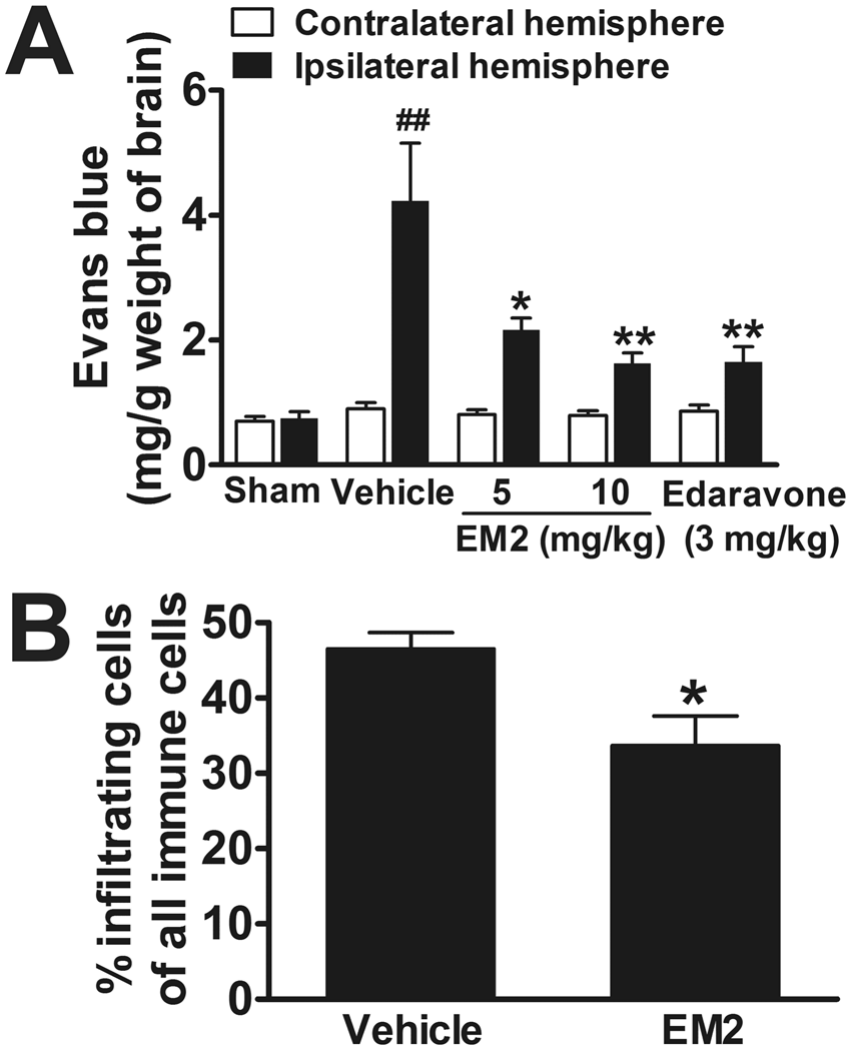
EM2 decreases the exudation of Evans blue and infiltrating immune cells in brains of I/R mice. (**A**) The amounts of blue dye in the hemispheres at 48 h after reperfusion were measured and compared among groups (n = 10). (**B**) Quantitative analysis of infiltrating immune cells (CD45^high+^) in the ipsilateral hemisphere following 72 h of reperfusion by flow cytometry (n = 4). All data are presented as means ± S.E.M. ^##^ *p* < 0.01 compared with sham group. *p < 0.05 and **p < 0.01 compared with vehicle group.

To detect whether EM2 has any effect on the BBB integrity, mice were subjected to Evans blue exudation analysis. In sham-treated mice, there was no significant difference of blue dye extravasations between the two hemispheres of brain (Fig. 5A). I/R injury destroyed the integrality of BBB and increased the permeability of BBB, as evidenced by the markedly increase of blue dye exudation in the ipsilateral (ischemic) hemisphere compared with contralateral (non-ischemic) hemisphere. EM2 significantly and does-dependently decreased the amount of Evans blue at both doses (5 and 10 mg/kg) (Fig. 5A), indicating that EM2 could attenuate I/R-induced BBB leakage.

Furthermore, we assessed the central nervous system (CNS) infiltration of peripheral (CD45^high+^) immune cells through BBB by flow cytometry analysis. We found that infiltration of CD45^high+^ immune cells was significantly reduced in the brains of 10 mg/kg EM2-treated mice compared to vehicle controls (Fig. 5B), which is consistent with previous report mentioning the immune cell infiltration was attenuated inEphA2-deficient mice^11^.

**Figure 6:**
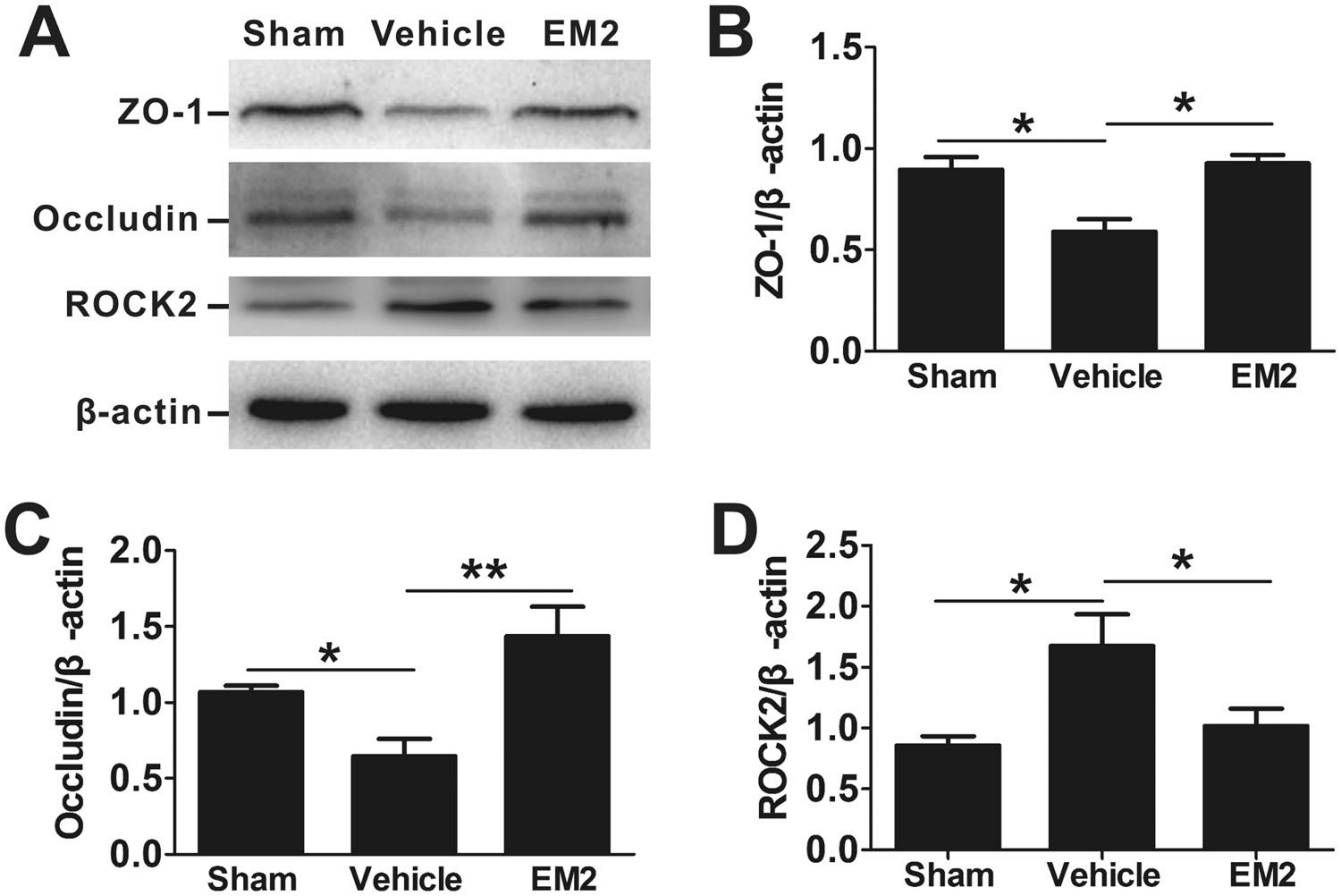
EM2 attenuates the disruption of tight junction proteins following focal cerebral I/R. (**A**) Western blot analysis for ZO-1, Occludin and ROCK2 in cerebral hemisphere after 24 h reperfusion. (**B**,**C** and **D**) Quantitation of ZO-1, Occludin and ROCK2 expression, respectively, normalized to β-actin. The data are presented as means ± S.E.M. (n = 4–5). *p < 0.05, **p < 0.01 compared with indicated group.

We also examined BBB damage by evaluating the levels of tight junction proteins, such as ZO-1 and Occludin. I/R injury induced reduction in expression of ZO-1 and Occludin, compared to the sham control (Fig. 6A–C). However, the reduction in ZO-1 and Occludin was prevented in EM2 (10 mg/kg)-treated mice (Fig. 6A–C), which improve the BBB integrity and may contribute to its BBB protective effect.

Activation of EphA2 receptor destabilizes adherens junctions through a RhoA-dependent mechanism^19^. Rho proteins function through Rho-associated protein kinase (ROCK1 and ROCK2)^20^, which up-regulates the expression of MMP-9^21^ and aggregates BBB injury. Therefore, we checked the expression of ROCK2, one of the two isoforms which is mainly expressed in neurons and vessels^22, 23^. We found that ROCK2 expression was significantly increased in the cortex of vehicle group (Fig. 6A and D). However, EM2 treatment (10 mg/kg) partially restored the expression of ROCK2 to a level similar to sham group. Therefore, the Rho/ROCK signaling pathway was involved in the BBB protective effect of EM2.

### Blockage of EM2 on EphA2 receptor activation

**Figure 7:**
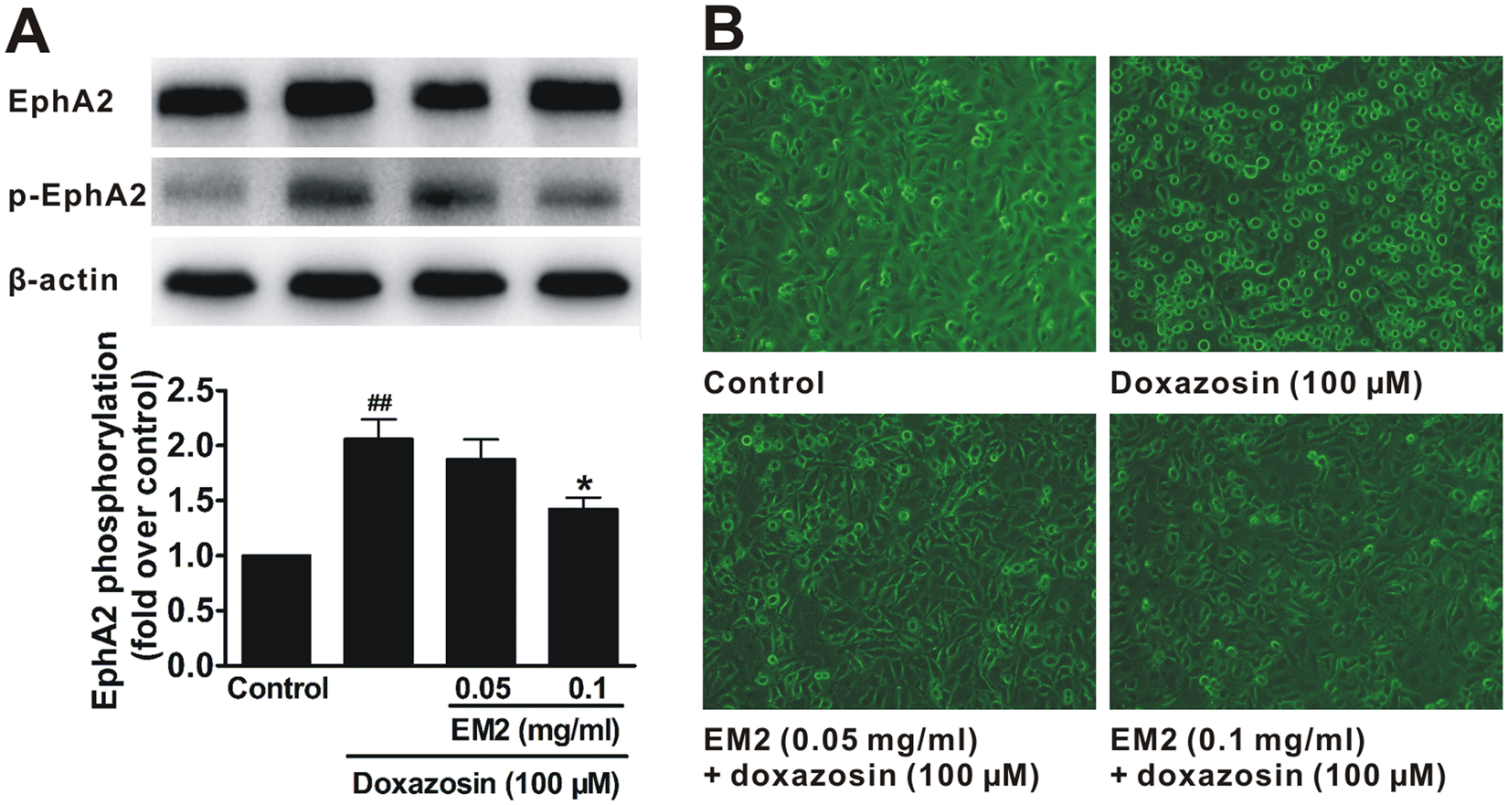
EM2 reduces EphA2 phosphorylation and the cell rounding caused by the agonist. (**A**) The protein level of p-EphA2 (phosphorylated EphA2). Representative western blotting analysis and the quantifications are shown on the top and bottom, respectively. Data are presented as means ± S.E.M. (n = 3). ^##^ *p* < 0.01 compared with control group. *p < 0.05 compared with doxazosin (agonist group). (**B**) Representative photographs of the morphological changes in PC3 cells.

PC3 cells highly express EphA2 receptors^24, 25^. To determine whether EM2 antagonizes EphA2, PC3 cells were used to observe the influence of EM2 on EphA2 phosphorylation induced by doxazosin, an agonist of EphA2. We found that the basal phosphorylation of EphA2 (p-EphA2) at Tyr588^26^ was at a low level in non-activated cells (Fig. 7A). In cells stimulated by doxazosin, the phosphorylation of EphA2 (p-EphA2) was increased. EM2 significantly reduced the amount of p-EphA2 at 0.1 mg/ml, and showed a trend of similar inhibitory effects at a lower concentration (0.05 mg/ml), suggesting that EM2 could inhibit doxazosin-induced EphA2 activation (Fig. 7A). In addition, cell morphological observation showed that doxazosin-treated cells became round losing their original shuttle shape, and the gap between cells increased (Fig. 7B). In contrast, the EM2 treatment at either low- (0.05 mg/ml) or high-dose (0.1 mg/ml) could prevent cells from doxazosin-induced rounding (Fig. 7B). These data demonstrated that EM2 shows EphA2 antagonistic activity in PC3 cells.

## Discussion

In this study, we prepared four mutant proteins of EphrinA1 with truncation and/or residues mutation. One of them, EM2, significantly reduced cerebral infarct size and neuronal apoptosis, improved neurological outcomes, alleviated cerebral edema, attenuated CNS peripheral immune cell infiltrates and decreased BBB leakage in a mouse focal cerebral I/R model. Such beneficial effects are attributed to the improved BBB integrity due to its antagonistic activity on EphA2.

Ischemic stroke causes BBB disruption, which further exacerbates brain injury by the development of cerebral edema and hemorrhagic transformation^27^. Protection of BBB from ischemic injury can alleviate brain damage and thus play a neuroprotective role. EphrinA1/EphA2 (ligand/receptor) signaling on vascular endothelial cells regulates integrity of endothelial cell junctions, resulting in vascular leakage^19, 28^. EphA2 deletion significantly reduces ischemia-induced BBB damage in mice^11^, and EphA2 inactivation promotes tight junction formation in HBMEC^12^.

The structure of EphrinA1/EphA2 complex has been clearly resolved^13^. EphrinA1 functions as an EphA2 agonist. Thus EphrinA1 was selected as a target protein to engineer EphA2 antagonist, which should have the potential of binding to EphA2 but not activate EphA2. EphrinA1 G–H loop is the EphA2-binding domain, which protruded from the EphrinA1 and poked into a channel of EphA2. After analyzing the EphrinA1 structure, we selected this crucial loop domain and its supporting scaffold to generate EM1. We then engineered other mutants based on EM1, by substituting all residues RFTPFTLGKEFK of this loop with SWLAYPGAVSYR (EM2), or minimizing the supporting scaffold (EM3), or making a single residue mutation (E54G) within the loop (EM4). As expected, all four mutants were structurally stable.

We further investigated the neuroprotective effects of these EphrinA1 mutants in a mouse model of stroke. Interestingly, we found that the mutants with EphrinA1 wild-type loop (EM1 and EM3) showed no neuroprotective effect. EM1 and EM3 may still serve as EphA2 agonist. On the other hand, mutants with substituted loop (EM2) or mutation in the loop (EM4) showed neuroprotective effects in different degrees, indicating indirectly that both EM2 and EM4 would be EphA2 antagonist. A single E54G mutation in EM4 converted the agonistic activity into minor antagonistic activity. This result provided additional evidence that E119 of EphrinA1 contributed its EphA2-activating function, which is consistent with previous study^13^. More importantly, EM2 exhibited a much stronger neuroprotective effect compared with EM4, suggesting a higher antagonistic activity of EM2. Therefore, amino acids other than E54 in the loop can also influence the activity of protein. These results demonstrate the importance of the residues in EphA2-binding loop, which determines whether the mutant is an agonist or an antagonis by triggering different biological responses of EphA2 receptor.

EM2 significantly reduced BBB permeability in mice subjected to cerebral I/R, as evidenced by reduced post-stroke edema, Evans blue extravasation and immune cell infiltrates. These results suggested that EM2 may protect BBB against destruction and thus contribute to the neuroprotective effect. We further investigated mechanisms related to the damage of the BBB by evaluating the levels of tight junction proteins, ZO-1 and Occludin, which are responsible for maintaining BBB integrity. We found that EM2-treated mice had significantly higher levels of ZO-1 and Occludin proteins. Moreover, the level of ROCK2 increased in mice with cerebral I/R injury, which can be substantially reduced by EM2 treatment. Therefore, EM2 may protect I/R-induced BBB damage through Rho/ROCK signaling pathway.

In addition, EM2 could markedly reduce agonist doxazosin-induced EphA2 phosphorylation and morphological change (cells rounding) in PC3 cells, which further confirmed the antagonistic activity of EM2 against EphA2.

In summary, we engineered four EphrinA1 mutants. Among them, EM2, exhibited significant neuroprotection in I/R model of mice, and this effect may be attributed to its BBB protection by antagonizing EphA2. Our results demonstrate that a receptor agonist can be converted to an antagonist by amino acid mutations, and the blockage of EphA2 activation could be a new approach for treatment of cerebral ischemic injury.

## Methods

### Bacterial strains, plasmids, animals and ethics statement

Plasmids pEASY-T1 and *E. coli* DH5α were obtained from TransGen Biotech (Beijing, China). pET-30a(+) and *E. coli* BL21(DE3) (Novagen, Madison, USA) were maintained in our laboratory. Male KM mice (28–30 g) were obtained from the Experimental Animal Center in Peking University Health Science Center. The experimental designs and all procedures were approved by the Committee on the Ethics of Animal Experiments in the Peking University Health Science Center (Permit Number: LA2013-69), in accordance with the National Institutes of Health guide (NIH Publications No. 8023, revised 1978).

### Homology modeling and molecular docking of EphrinA1 mutants

Computer modeling and protein-protein docking were carried out using Discovery Studio 2016 (BIOVIA, San Diego, USA). Four EphrinA1 mutants (EM1, EM2, EM3, and EM4) were designed. The structure templates for modeling were found via BLAST. EM1, EM3, and EM4 were modeled based on the structure of EphrinA1 (PDB ID: 3HE1) as a template. EphrinA1 and other proteins (PDB ID: 2VSY, 3MPC, 2CIB, 4RG3) were combined as the template for EM2. Homology model was constructed using the program MODELLER^29^. Then, the obtained model structures were evaluated with the Ramachandran plot^30^ and Profiles-3D programs^31^.

Protein-protein docking program ZDOCK^32^ was used to analyze the interaction between EphrinA1 mutants and EphA2 receptor. Angular step size of ligand orientation was set to 6, generating a total of 54,000 poses in total. After filtered by the amino acids of interaction interface, these poses were further analyzed by RDOCK program^33^. The poses with best-score and lowest-energy were selected as interaction models.

### Construction and expression of recombinant proteins

The nucleotide sequences that code the four proteins were generated by PCR cloning. NdeI and BamHI sites were appended to the 5′- and 3′-ends of each DNA fragment, respectively. PCR products were cloned into TA cloning vector pEASY-T1 and sequenced. The DNA fragments were sub-cloned into pET-30a(+) plasmids by NdeI and BamHI sites. All plasmids were verified by DNA sequencing. Then these recombinant expression plasmids (pET30a-EM1, pET30a-EM2, pET30a-EM3 and pET30a-EM4) were transformed into *E. coli* BL21 (DE3), which were grown at 37 °C to OD_600_ = 0.6 in LB media containing 100 mg/ml kanamycin and induced with isopropyl-b-D- thiogalactoside (IPTG).

### Refolding and purification of recombinant proteins

Bacteria expressing recombinant proteins were harvested and re-suspended in pre-cooled buffer A (50 mM Tris-HCl, 0.1 mM EDTA, 100 mM NaCl, 5% glycerol, pH 7.9) containing 1% Triton X-100. After sonication and centrifugation at 12,000 × g for 10 min, the pellets containing EMs proteins (in the form of inclusion bodies) were washed twice by purgation buffer (50 mM Tris-HCl, 1 M urea, pH 7.9), and then resuspended in lysis buffer (50 mmol/L Tris-Cl, 8 M urea, 150 mM NaCl, pH7.9) at 25 °C overnight. The supernatants containing denatured proteins were collected by centrifugation at 12,000 × g for 10 min. The renaturation of inclusion bodies was performed with dilution method^34^. Briefly, inclusion bodies (10 ml, 0.2 mg/ml) were diluted by adding same volume of glycerol, and then quickly added to 200 ml renaturation buffer (20 mM Tris-HCl, 0.5 mM reduced glutathione, 0.5 mM oxidized glutathione, 0.5 mM L-argine, pH 7.9) with rapid agitation for 2 h at room temperature, and followed by slow agitation for 24 h at 4 °C.

Refolded proteins were collected by centrifugation at 12,000 × g for 10 min, and purified through affinity chromatography via His-tag at the C-terminus. Protein samples were applied, respectively, to individual pre-equilibrated column packed with 5 ml Ni ^2+^ NTA resin (Invitrogen, USA). The columns were washed with five times the column volumes of binding buffer (50 mM Tris-HCl, 500 mM NaCl, 20 mM Imidazole, pH 7.9), and then eluted with stepwise gradient elution buffer (50 mM Tris-HCl, 500 mM NaCl, pH 7.9; the concentration of Imidazole was increased stepwisely from 50 to 100, 150, 200, 300 and 500 mM). The eluted fractions were collected and analyzed on a 15% SDS-PAGE. Fractions containing target proteins were pooled and dialyzed against deionized water at 4 °C thoroughly, and then followed by lyophilization and storing at −80 °C.

### Middle cerebral artery occlusion (MCAO) model in mice and treatment

Mice MCAO model was prepared as previously described^35^. Briefly, mice were anaesthetized with chloral hydrate (500 mg/kg, i.p.), then a nylon filament with blunted tip coated with silicone oil was introduced into detached internal carotid artery from the external carotid artery to occlude MCA. After 1.5 h occlusion, the monofilament was gently removed, and the blood supply was restored. Mice were treated with vehicle (normal saline, NS), EM1, EM2, EM3, EM4 or edaravone (positive control). Vehicle and proteins were administered immediately at the end of occlusion with a single injection (i.v.). Edaravone was injected (3 mg/kg, i.v.) twice at the beginning and end of occlusion, respectively, according to previous published works^36, 37^.

### Determination of neurological deficit, infarct volume and edema

Neurologic deficit was evaluated after 24 h reperfusion in a blinded manner as previously described^38^. Then, mice were deeply anesthetized by chloral hydrate (500 mg/kg, i.p.) and transcardially perfused with NS. The wet weight of the isolated brain was measured. Then, the brain was sliced coronally into 5 pieces at 2-mm intervals and stained by immersing the slices into 1% TTC. The slices were fixed by 4% paraformaldehyde and photographed, and the normal and infarct areas were analyzed with ImageJ software (NIH Image, available on the Internet at http://rsb.info.nih.gov/nih-image/). The percentage of infarct volume was calculated using the method described previously^18^. Then these these slices were dried at 110 °C and their dry weights were examined. Water content was determined as follows: brain water content (%) = (wet weight – dry weight)/wet weight × 100%^39^.

### Terminal deoxynucleotidyl transferase-mediated dUTP nick end labeling (TUNEL) staining

Mice were anesthetized with chloral hydrate (350 mg/kg, i.p.) and transcardially perfused with 4% paraformaldehyde after 24 h reperfusion. Their brains were isolated and immersed in dry ice precooled isopentane. Coronal sections of 10 μm thickness were sliced with a cryostat and mounted on slides. Cryosections were stored at −20 °C. TUNEL assay was performed with an *in situ* apoptosis detection kit (KeyGen Biotech, Nanjing, China). Brain sections were incubated with a TUNEL mixture containing 0.5 U/μl TDT, 0.2 nmol biotin-11-dUTP, followed by streptavidin-FITC. After capturing images with a fluorescence microscope (Olympus IX71), quantification was performed by a blinded observer.

### Caspase-3 activity assay

The brains of anesthetized mice were isolated after 24 h reperfusion, and the proteins of ischemic hemispheres were extracted by homogenization. Protein concentration was determined by BCA assay (Pierce, Rockford, USA) and adjusted to 2 μg/μl. The activity of caspase-3 was detected using a Caspase-3 Colorimetric Assay Kit (KeyGen Biotech, Nanjing, China). 100 μg proteins were mixed with 50 μl of 2 × Reaction buffer supplied with 5 μl of Caspase-3 substrate then incubated at 37 °C in dark for 4 h. The activity of caspase-3 was quantified spectrophotometrically at 405 nm wavelength in a microplate reader (Thermo Scientific, Waltham, MA, USA), and presented as the increase fold over the sham group.

### Measurement of Evans blue exudation

The experiment was performed as described previously^39^. MCAO model was prepared and animals were treated as above mentioned. Anesthetized mice received Evans blue (80 mg/kg, i.v.) 3 h before sacrifice (48 h after reperfusion), and then were transcardially perfused with NS to washout the intravascular blood and the dye. Evans blue dye in collected brain tissues was extracted with formamide (1 ml) at 45 °C for 72 h, which was detected with spectrophotometer at 610 nm wavelength. The concentration of Evans blue was calculated according to the standard curve. Left and right hemispheres were dried at 110 °C for 8 h and weighed, respectively. The dye content in brain was calculated as the following formula: exudative amount of Evans blue (mg/g) = W1/W2, wherein W1 and W2 represent the amount of dying material and the dry weight of brain hemisphere, respectively.

### Flow Cytometry

Flow cytometric analyses were performed to detect the immune cells in brains as previously described^11, 40^. Mice were anesthetized and transcardially perfused with cold PBS after 72 h reperfusion. Their brains were collected and roughly homogenized in PBS, resuspended in PBS containing 3 mg/ml collagenase D (Roche Diagnostics, Indianapolis, IN, USA) and 0.1 mg/mL DNAse I (Roche Diagnostics, Indianapolis, IN, USA), and incubated for 30 minutes at 37 °C. These brain homogenates were filtered through cell strainers (70 μm; BD Biosciences), centrifuged (10 minutes, 2,000 rpm), and collected. Then the cells were incubated with standard erythrocyte lysis buffer. Cells were washed and resuspended in PBS, and stained with CD11b and CD45 antibodies (BD Biosciences, San Jose, CA, USA) (30 minutes, room temperature), and then analyzed using flow cytometry (Gallios, Beckman Coulter, CA, USA).

### Cell culture and treatment

The human prostatic carcinoma cell line, PC3, was cultured in Dulbecco’s modified Eagle’s Medium (DMEM, M&C Gene Technology, Beijing, China) supplemented with 10% fetal bovine serum (FBS, Gibco, Australia) and 0.05 U/mL penicillin and 0.05 mg/ml streptomycin at 37 °C with humidified 5% CO_2_ and 95% air. The cultured medium was changed twice a week.

Cells were plated onto 6-well plates and incubated until 70–80% confluency, then the regular medium was replaced with serum-free medium (DMEM) overnight. The *in vitro* experiment was divided into four groups: Control (0.1% DMSO treated), doxazosin (100 μM; J&K, Beijing, China), doxazosin (100 μM) + EM2 (0.05 mg/ml), doxazosin (100 μM)+ EM2 (0.1 mg/ml). Cells were treated with indicated agents for 1 h.

### Western blotting analysis

The proteins of cultured cells and brain cortices were extracted by mixing with RIPA buffer (50 mM Tris-HCl pH7.4, 150 mM NaCl, 2 mM EDTA, 0.1% SDS) supplemented with 1 mM serine protease inhibitor phenylmethanesulfonyl fluoride (PMSF) (Sigma-Aldrich, St. Louis, MO) and 1% phosphatase inhibitor mixture P1260 (Applygen Technologies Inc., Beijing, China) and kept on ice for 30 min. The homogenates were centrifugated at 12,000 × g at 4 °C for 15 min. The protein concentrations of the supernatants were determined by the BCA assay (Pierce, Rockford, USA). Denatured samples were separated by SDS–PAGE, and blotted onto the PVDF membrane (Millipore, Billerica, MA, USA) using a wet transfer system (Bio-Rad, Hercules, CA, USA). The membrane was immersed in blocking solution (5% milk, 20 mM Tris-HCl, 0.1% Tween 20, pH 7.4) for 2 h at room temperature, and then incubated with a monoclonal rabbit anti-phospho-EphA2 (Tyr588) (1:1000, Santa Cruz, CA, USA), or a polyclonal rabbit anti-EphA2 (C-20) antibody (1:1000, Santa Cruz, CA, USA), or a polyclonal rabbit anti-ZO-1 antibody (1:1000, abcam, MA, USA), or a polyclonal goat anti-Occludin (C-19) antibody (1:200, Santa Cruz, CA, USA), or a polyclonal rabbit anti-ROCK2 antibody (1:1000, bioss, Beijing, China), or a rabbit anti-β-actin antibody (1:2000, Santa Cruz Biotechnology, CA, USA) overnight at 4 °C. The membranes were incubated with a horseradish peroxidase-labeled goat-anti-rabbit or rabbit-anti-goat secondary antibody (1:5000, Santa Cruz Biotechnology, CA, USA) at room temperature for 2 h, followed by ECL detection (Pierce, Rockford, IL, USA) and analyzed using ImageLab software (Bio-Rad, Hercules, CA, USA).

### Statistical Analysis

All values are presented as mean ± S.E.M. Statistical analyses were performed using the two-way ANOVA, followed by Scheffe’s post hoc test. *p* < 0.05 was considered statistically significant.

## Electronic supplementary material

Figure S1. Generation of the EphrinA1 mutants.
