# Supplementary material for: Design and evaluation of EphrinA1 mutants with cerebral protective effect
Source: Sci Rep. 2017 May 15;7:1881. doi: 10.1038/s41598-017-02091-7 (PMC5432519; doi:10.1038/s41598-017-02091-7)
Supplement: Supplementary file 1 — Figure S1. Generation of the EphrinA1 mutants. [file 41598_2017_2091_MOESM1_ESM.pdf]

Design and evaluation of EphrinA1 mutants with cerebral protective effect

Yuanjun Zhu[1,#], Yuanqing Gao[1,#], Danping Zheng[1,#], Mengyang Shui[1], Kuai Yu[1], Xiaoyan liu[1], Yuan Lin[2], Li Su[3], Wenxing Yang[4], Yinye Wang[1]

[1] Department of Molecular and Cellular Pharmacology, School of Pharmaceutical Sciences, Peking University Health Science Center, Beijing, China

[2] State Key Laboratory of Bioactive Substances and Function of Natural Medicine, Institute of Materia Medica, Chinese Academy of Medical Sciences and Peking Union Medical College, Beijing, China

[3] Center of Medical and Health Analysis, Peking University Health Science Center, Beijing, China

[4] Department of Organismic and Evolutionary Biology, Center for Brain Science, Harvard University, Cambridge, MA, USA

Correspondence and requests for materials should be addressed to Y.W. (wangyinye@bjmu.edu.cn) or Y.Z. (zhuyuanjun@bjmu.edu.cn).

[#] These authors contributed equally to this work.

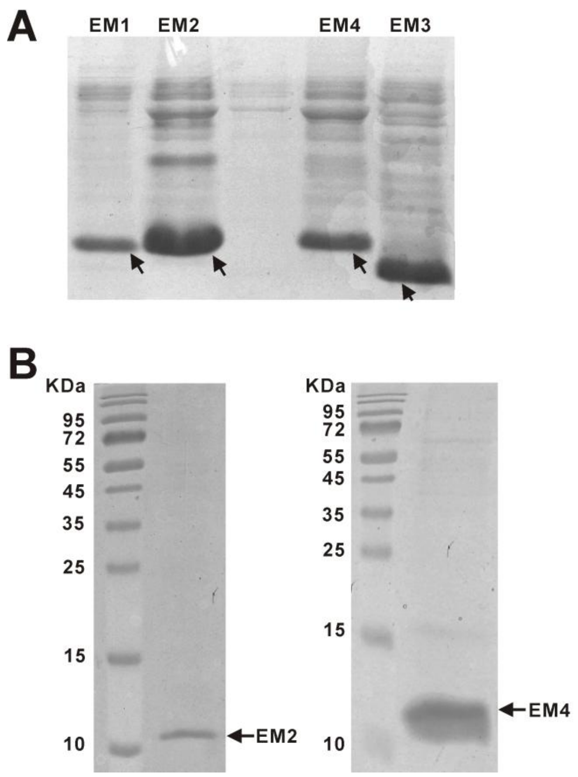

Figure S1. Generation of the EphrinA1 mutants. (A) Expression of recombinant proteins EM1, EM2, EM3 and EM4 from bacteria. *E. coli* BL21(DE3) cells with expression plasmids were induced as described in Methods. These proteins were expressed in inclusion bodies, as indicated by the arrows. (B) Purified EM2 and EM4 proteins using Ni-NTA affinity chromatography.
